# Hot Melt Extrusion as an Effective Process in the Development of Mucoadhesive Tablets Containing *Scutellariae baicalensis radix* Extract and Chitosan Dedicated to the Treatment of Oral Infections

**DOI:** 10.3390/ijms24065834

**Published:** 2023-03-19

**Authors:** Magdalena Paczkowska-Walendowska, Andrzej Miklaszewski, Daria Szymanowska, Krystyna Skalicka-Woźniak, Judyta Cielecka-Piontek

**Affiliations:** 1 Department of Pharmacognosy, Poznan University of Medical Sciences, Rokietnicka 3, 60-806 Poznan, Poland; 2 Institute of Materials Science and Engineering, Faculty of Materials Engineering and Technical Physics, Poznan University of Technology, Jana Pawła II 24, 61-138 Poznan, Poland; 3 Department of Biotechnology and Food Microbiology, Poznan University of Life Sciences, 48 Wojska Polskiego Street, 60-627 Poznan, Poland; 4 Department of Natural Products Chemistry, Medical University of Lublin, ul. Chodźki 1, 20-093 Lublin, Poland

**Keywords:** hot melt extrusion, *Scutellariae baicalensis radix*, chitosan, HPMC, controlled release

## Abstract

Hot Melt Extrusion (HME) technology was developed to obtain blends containing lyophilized *Scutellariae baicalensis* root extract and chitosan in order to improve the rheological properties of the obtained blends, including tableting and compressibility properties. (Hydroxypropyl)methyl cellulose (HPMC) in 3 different ratios was used as amorphous matrix formers. The systems were characterized using X-ray powder diffraction (PXRD), Fourier Transform Infrared Spectroscopy with Attenuated Total Reflectance (FTIR-ATR), and in vitro release, permeability, and microbiological activity studies. Then, the extrudates were used to prepare tablets in order to give them the appropriate pharmaceutical form. HPMC-based systems released baicalin more slowly, resulting in delayed peaks in the acceptor fluid. This behavior can be explained by the fact that HPMC swells significantly, and the dissolved substance must have diffused through the polymer network before being released. The best tabletability properties are provided by the formulation containing the extrudate with lyophilized extract HPMC 50:50 *w*/*w*. These tablets offer a valuable baicalin release profile while maintaining good mucoadhesive properties that condition the tablet’s retention in the application site and the effectiveness of therapy.

## 1. Introduction

Periodontal disease and other oral infections are a significant global burden on oral health, with severe periodontitis responsible for losing many teeth in the adult population worldwide. The WHO Global Oral Health Status Report (2022) estimated that oral diseases affect nearly 3.5 billion people worldwide. At the same time, severe periodontal disease is estimated to affect approximately 19% of the world’s adult population, equivalent to more than 1 billion cases worldwide [1]. Over the past 50 years, both systemic and local administration have been crucial methods for drug delivery to treat oral infections. The use of systemic administration in treating oral infections has yielded some positive results and has been widely used for antibiotic therapy in the treatment of periodontitis [2]. However, systemic administration may result in issues including dysbacteriosis and inadequate biodistribution [3]. Because of these apparent disadvantages of systemic administration, there is a great need for local drug delivery systems to improve the prevention and treatment of periodontitis and other oral infections. Local drug delivery systems that are placed directly on the oral mucosa can provide a sufficiently high concentration of an active substance for a reasonably long period of time. Other significant advantages of local drug delivery systems include avoidance of gastrointestinal problems and first-pass metabolism due to direct application to a specific site; higher efficacy and fewer side effects due to controlled drug release; and improved patient compliance due to reduced dosing frequency and easier oral application and the ability to quickly remove the drug in the event of irritability [4]. Moreover, local drug delivery systems exert therapeutic effects mainly through the content of three types of active compounds, including antibacterial agents, modulators of inflammation, and alveolar bone and tissue repair agents for treating periodontitis.

One of the medicinal raw materials for which much has been described and evidence of medical use exists is *Scutellariae baicalensis radix* (Baikal Skullcap Root). The healing effect of *S. baicalensis* root is due to the presence of bioactive compounds, mainly flavones such as baicalin and wogonoside and their aglycones, baicalein and wogonin [5]. Thanks to the content of bioactive compounds, primarily baicalin, the plant material has anti-inflammatory properties by inhibiting the expression of proinflammatory mediators such as IL-1, IL-6, IL-8, and TNF in gingival tissues, antioxidant properties, and antibacterial properties against *Streptococcus mutans*, *Fusobacterium nucleatum, Aggregatibacter actinomycetemcomitans*, and *Porphyromonas gingivalis* for treating and preventing oral disease [6,7]. In addition, it has been shown that baicalein can increase the expression of osteogenic markers in human periodontal ligament cells, which is valuable in treating periodontitis [8].

Despite the many valuable health-promoting properties of *S. baicalensis* root, the solubility of baicalin is limited, which classifies it as IV BCS [9]. The low solubility limits the application of compounds also in local drug delivery systems. Therefore, one of the biggest challenges for pharmaceutical researchers has been increasing the solubility of the insoluble compound with pharmacological potency. Various methods have been developed, including particle size reduction, solubilization, and solid dispersion, with the latter, produced by hot melt extrusion (HME) becoming increasingly desirable [10,11]. Compared to traditional techniques, HME can offer numerous advantages, both economic benefits due to the shorter time to manufacture the final product and environmental benefits due to the elimination of solvents in the processing process [12]. From a pharmaceutical process point of view, HME involves pumping polymeric materials with a rotating screw at temperatures above their glass transition temperature to achieve molecular-level mixing of active compounds and thermoplastic binders, polymers, or both. The components are changed by this molecular mixing into an amorphous product with a homogeneous shape and density, which improves the dissolution profile of the poorly water-soluble substance [13].

As mentioned above, the low solubility of baicalin limits its application. Therefore, this work aimed to use HME technology to improve the physicochemical properties of baicalin as concluded in lyophilized *S. baicalensis* root extract. Nevertheless, the use of the extrudate itself in medicine is limited; hence, the optimization of the tableting process was done. Thus, the impact of the HME process on the rheological properties, such as tableting and compressibility properties, of the obtained extrudate-based blends was additionally assessed. Receiving appropriate baicalin release profiles as well as mucoadhesive functionality was indicated as being necessary for the development of this form of the drug.

## 2. Results and Discussion

In the first stage, a lyophilized extract of *Scutellariae baicalensis radix* was obtained according to the procedure described previously [14]. In an earlier study, the phytochemical and biological properties (including antioxidant and anti-inflammatory activity) of the obtained extract were confirmed. At the same time, the work aimed to use HME to improve the parameters for the release of active compounds from the obtained tablets and also to improve the tableting process itself. For a better understanding of all experimental work, all steps were collected in Figure S1 (Supplementary Materials).

As the first task, preparing three types of solid dispersions were possible using the hot melt extrusion technique. Importantly, in all cases, the torque measured during extrusion was similar (around 0.72 Nm), not causing any difficulty during processing. The process temperature (150 °C) did not decompose the active ingredient, i.e., baicalin (melting point 202–205 °C). This was also checked during the evaluation of the content of active compounds by the HPLC method (chromatogram of standards presented in Figure S2, Supplementary Materials), which was validated according to ICH guidelines and whose validation parameters are collected in Table S1 (Supplementary Materials). The phytochemical profile remained at the same level as in the original lyophilized extract: baicalin—2.61 mg per 100 mg of extract; baicalein—323.40 µg per 100 mg of extract; and wogonin—40.30 µg per 100 mg of extract. With the preserved phytochemical profile of bioactive compounds, there was no need to re-examine the biological properties because it is the content of active compounds that determines those effects.

**Figure 1:**
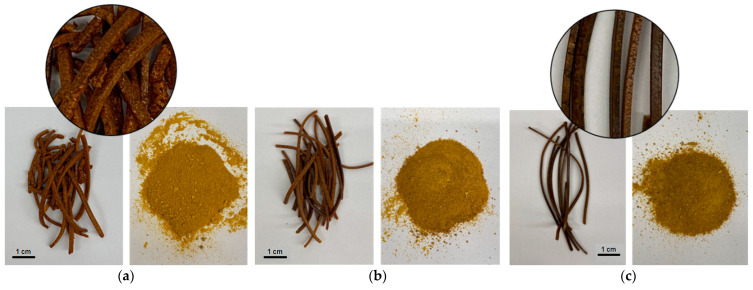
Macroscopic pictures of hot melt extrudates (surfaces of blended and grounded) based on HPMC in three ratios: (**a**) system-HPMC 75:25 *w*/*w*; (**b**) system-HPMC 50:50 *w*/*w*; (**c**) system-HPMC 25:75 *w*/*w*.

Figure 1 shows the macroscopy pictures of HPMC-based extrudates. As seen, in the case of system-HPMC 75:25 *w*/*w* the inner structure appeared to be non-homogeneous, with a slightly rough surface and somehow “granular” inner structure with variable color. The internal structure changed as the amount of HPMC in the extrudate increased. So in the case of system HPMC 25:75 *w*/*w* outer structure appeared to be rather homogeneous and the surface relatively smooth.

The obtained extrudates were characterized by their structure (XRPD) and possible intermolecular chemical bond formation (FTIR-ATR).

**Figure 2:**
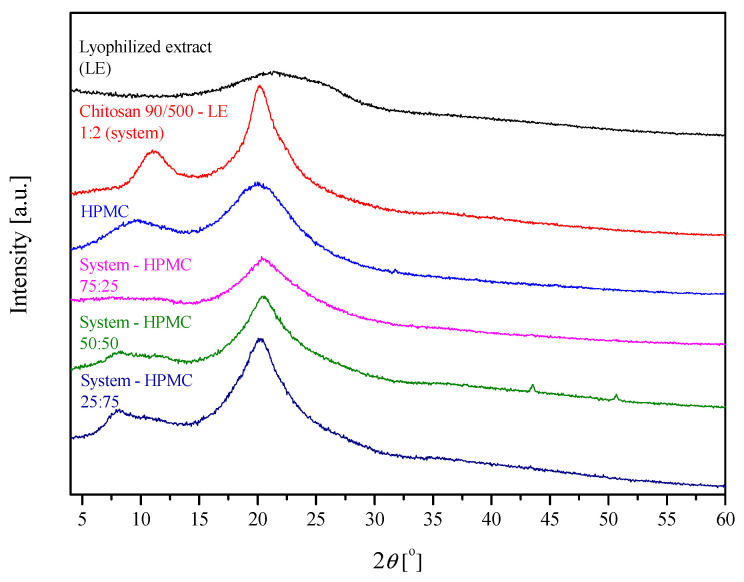
Diffractograms of extrudates.

The X-ray diffractograms (Figure 2) of the lyophilized extract and its system with chitosan show a large broadening of the diffraction peaks, which at low intensity indicates their amorphous structure, which was described previously [14]. HPMC is also amorphous in nature. So, hot melt extrudates are based on HPMC, which can be named an amorphous matrix former, transformed into an amorphous state, or molecularly dissolved in the carrier [15,16]. It is shown that the relationship in the 75/25 system shows the lowest degree of order (the lowest intensity and visibility of reflections). The increase in the amount of HPMC in the relationship with chitosan results in a clearer structural response of the extrudate system (an increase in the intensity of reflections and their visibility)—indirectly, this indicates a better reaction of the extract with chitosan to obtain an amorphous system. However, the addition of HPMC is necessary for processing reasons. The addition of the extract affects the position of reflections in the obtained extrudates. From the analysis carried out for the systems, the averaged result based on the chitosan base gives a reduction in the interplanar distances. However, no linear relationship was noted in all analyzed cases (Table 1).

**Figure 3:**
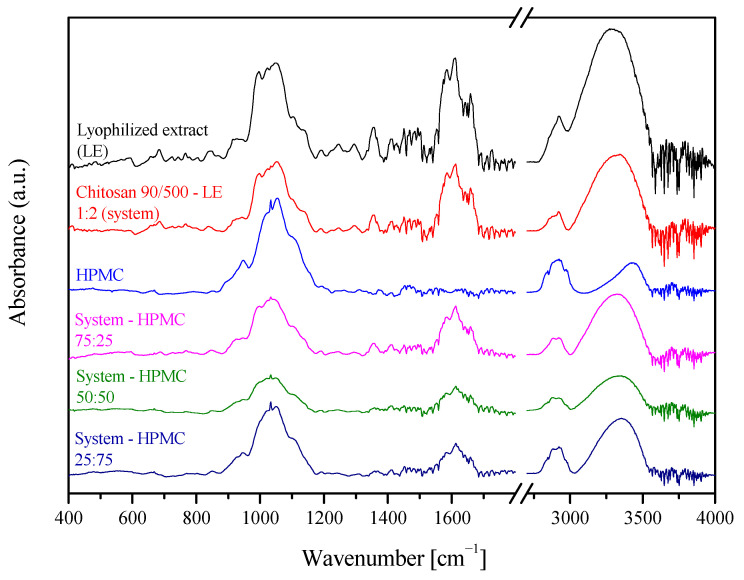
FTIR-ATR spectra of extrudates.

The obtained extrudates were characterized by their possibility to form intermolecular chemical bonds (FTIR-ATR) (Figure 3). Bands of *S. baicalensis* lyophilized extract at 3330 cm^−1^, 1720 cm^−1^, and 1660 cm^−1^ are characteristic for vibration of the O–H, –COOH, and C=O groups, while signals at 1600 cm^−1^ and 1580 cm^−1^ for the C=C vibration of the aromatic rings in the structure of flavones. The broad bands in the range 1200–900 cm^−1^ are characteristic of vibrations of C–O bonds of saccharides [14]. For the HPMC spectrum, a wide band was observed at 3300 cm^−1^, associated with the presence of -OH groups. While the complex band between 1200 and 950 cm^−1^ is related to numerous C–O vibrations, including glycosidic C-O-C, C-OH, C-OCH_3_, C-OCH_2_CH_2_OH [17]. In the case of extrudates, it can be observed that the bands at 3300 and 1600 cm^−1^ changed, broadened, and decreased in intensity, which means intermolecular hydrogen bonds between the extract and carrier, which has also been observed for solid dispersions of pure baicalin [18].

**Figure 4:**
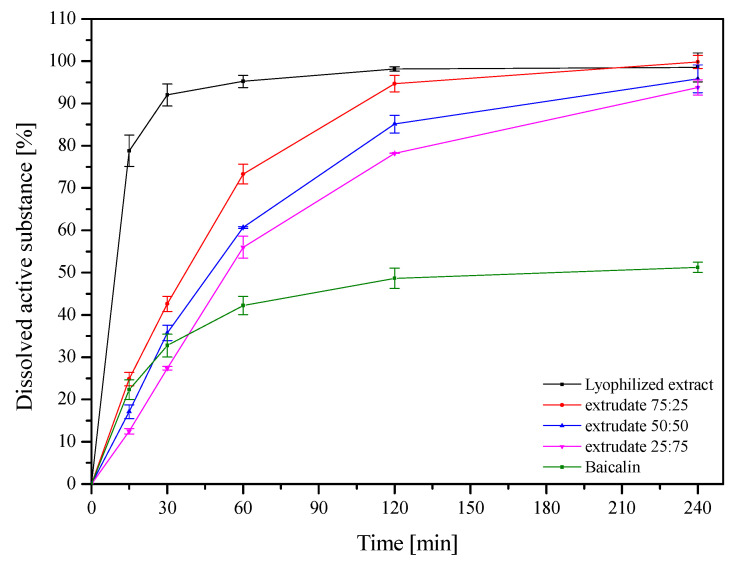
Dissolution profiles of baicalin from the lyophilized extract and extrudates (*n* = 6).

Figure 4 shows the release profiles of baicalin from ground hot melt extrudates based on HPMC in three different ratios. For comparison, the baicalin release from the lyophilized extract as well as the dissolution rate of pure baicalin are also shown. The dissolution rate of pure baicalin was very low; only 50% of pure baicalin was dissolved in 4 h due to its poor wettability and agglomeration. An increased dissolution rate of baicalin from the freeze-dried extract was observed, reaching 80% dissolved baicalin within 15 min, which is related to the change from crystalline to amorphous form. The HME process additionally improved the dissolution. Despite the slower solubility of baicalin, 80% over 90 min due to the presence of HPMC, the HME process improves wettability, reduces the size of baicalin dispersion, and prevents agglomeration of particles [19]. Differences in baicalin release from HPMC extrudates depend on the amount of carrier in the system. Firstly, differences in dissolution rates were statistically significant among the three extrudates (in all cases, *f*_1_ was below 20 and *f*_2_ was below 50). Secondly, with the increase in the amount of HPMC, baicalin dissolves to a lesser extent due to the hydration of the outer layer of the system, which causes the formation of a gel layer on its surface. This reduces the amount of water that enters the system’s core, which can hinder the movement of the active compounds and cause them to dissolve slowly [20].

Additionally, permeability coefficients using the PAMPA test were established. While the permeation test is not critical when talking about local application, it is intended to more extensively check the material’s properties after extrusion. The permeability coefficient for pure baicalin, calculated from equation no. 1 (*n* = 6), was 0.02 ± 0.01 × 10^−6^ cm/s, which is in line with previous research [21], and also confirms its low permeability, classifying baicalin as BCS IV [9]. Due to the improved solubility associated with the amorphization of baicalin, the permeability of the compound also increased. Permeability coefficients for extrudates were 0.96 ± 0.02, 0.74 ± 0.02 and 0.58 ± 0.02 × 10^−6^ cm/s, respectively, for extrudates 75:25, 50:50 and 25:75. The decrease in the permeation coefficient with the increase in the HPMC content in the system can also be explained by the formation of a gel layer, which makes it difficult for the active ingredients to reach the biological barrier. However, thanks to the amorphization of the system, multiple increases in the permeation of baicalin can be observed, which in turn is associated with an increase in its dissolution rate from extrudates. Nevertheless, penetration above 1 × 10^−6^ cm/s was still not achieved, so it cannot be said that the system penetrates well. What is intended from the point of view of topical application within the oral cavity, a very well-constructed system has been achieved where baicalin appears at the application site in a higher dose due to the increase in release rate while not penetrating the systemic circulation, staying at the place of application.

**Figure 5:**
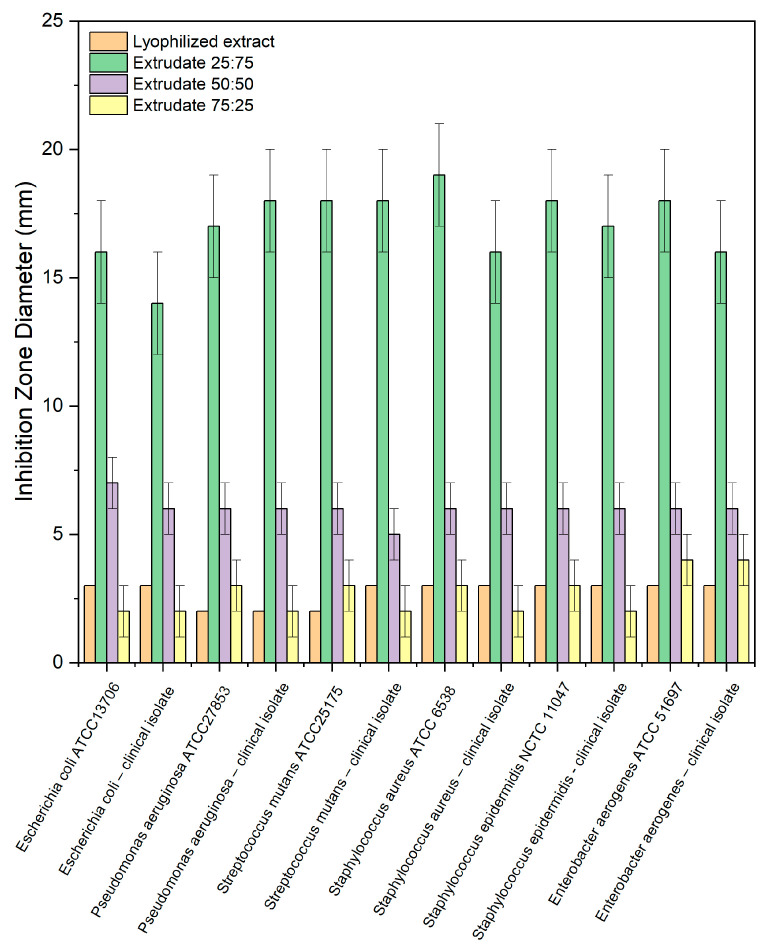
Influence of the lyophilized extract and its extrudates on indicator microorganisms (reference strains and clinical isolates) by the well-diffusion method.

In the presented study, the antimicrobial activity of the prepared extrudates against microorganisms colonizing the oral cavity (e.g., *S. mutans*) and bacteria whose presence in the oral cavity causes the development of infection (e.g., *P. aeruginosa. S. aureus, E. aerogenes*) was investigated. The antimicrobial activity of binary systems was evaluated according to their inhibition zone diameter against six species of bacteria (Figure 5). Table 2 presents the results of the impact of those tested on the ability to increase micro- organisms in the medium. Apart from the obvious fact that the lyophilized extract has antibacterial activity, which was described earlier, it is worth noting that chitosan has an equally important effect on the activity of the extrudates. So, the results revealed the highest antimicrobial activity for extrudate 25:75. In contrast, the most significant increase in activity following the combination of *S. baicalensis radix* extract with chitosan was observed against *S. mutans,* the most sensitive strain and, at the same time, one of the pathogens causing periodontitis. In liquid cultures, three different concentrations of extrudates were used. The results in Table 2 shows that only a concentration of 100 mg/mL exhibited antimicrobial activity.

In the next step, tablets containing all three types of extrudates were successfully prepared. A total of 6 formulations were prepared: three had extrudates (formulations F1, F3, and F5) and three contained the identical amounts of ingredients but in the form of powders, which were controls and comparative formulations (formulations F2, F4, and F6). HPMC was a carrier for hot melt extrusion in all formulations. Still, it also imparted mucoadhesive properties to the prepared systems.

**Figure 6:**
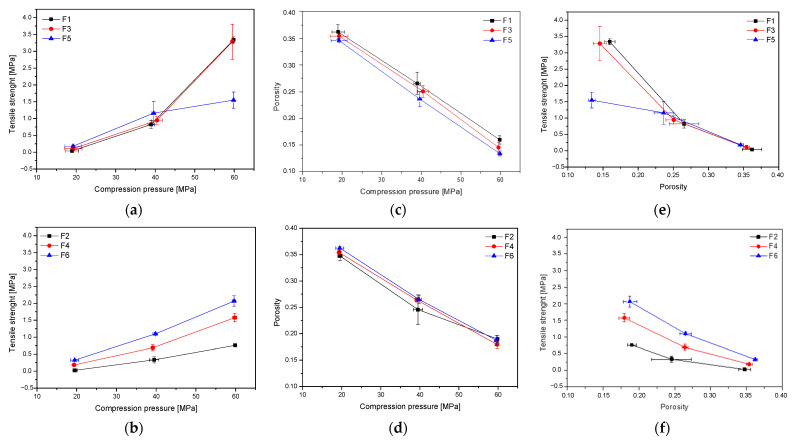
Tabletability (**a**,**b**), compressibility (**c**,**d**) and compactibility (**e**,**f**) of the extrudates and powder systems.

Firstly, tablets containing formulations F1–F6 were initially characterized in terms of tabletability, compressibility, and compactability (Figure 6). The tabletability of the tablets decreased in the following order: F1~F3 > F5 > F6 > F4 > F2 (Figure 6a,b). Such an order is related to the composition of the individual components. In general, it can be said that extrudate-based tablets showed better tabletability properties than those containing powders. However, a smaller amount of HPMC in the tablet increases its tabletability. According to the overall trend of the compressibility profile, the porosity level or the solid fraction value decreases as the pressure load applied to the powder samples grows (Figure 6b). The compactibility profiles for all six formulations are generally comparable, with little benefit for powder-based tablets. Finally, a powder’s compactability is defined as its ability to form coherent, strong tablets. Low-density tablets are obviously more porous because they have more pores, leading to poorer interparticle bonding. As a result, less power is needed to break down those tablets. The order of decreasing compactability appears to be as follows: F1 > F3 > F6 > F5 > F4 > F2 (Figure 6d,e). It can also be seen from Figure 6c that tablet tensile strength decreases exponentially with increasing porosity, which fits the Ryshkewitch equation (equations no. 5–6) where T_S0_ is the extrapolated tensile strength at zero porosity and T_S0_ is often used to compare bond strength. The T_S0_ of F2, F4, and F6 was, respectively, 1.5, 2.4, and 3.2 Mpa, the bonding strength of powders was poor. The T_S0_ of extrudates were enhanced to 4.7 and 4.2 for F1 and F3, and obtained a surprisingly low value for F6–1.7 Mpa. Bond strength increased, illustrating that the HME process can significantly improve bond strength. This may be due to better uniformity and closer contact during melting and extrusion. It can also be the result of the transformation from the crystalline to the amorphous form of the active compounds [19]. Based on the above parameters, the best tablet properties were obtained for formulations F1 (extrudates of system-HPMC 75:25) and F3 (extrudates of system-HPMC 50:50).

**Figure 7:**
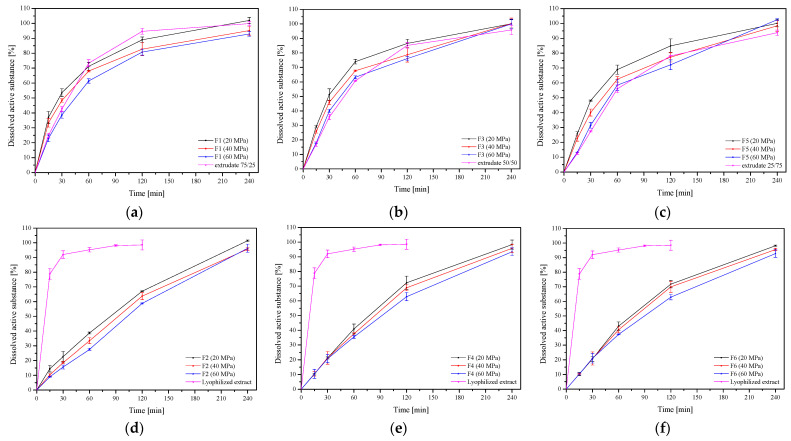
Dissolution profiles of baicalin from the extrudate-based tablets (**a**–**c**) and the powder-based tablets (**d**–**f**) (*n* = 6).

**Figure 8:**
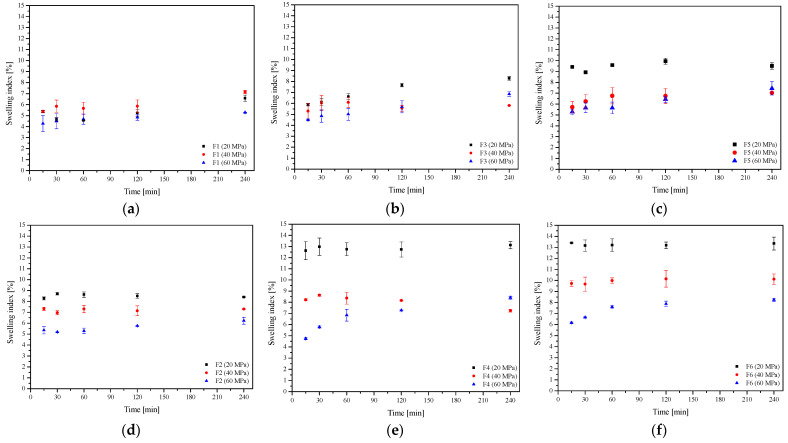
Swelling index of the extrudate-based tablets (**a**–**c**) and the powder-based tablets (**d**–**f**) (*n* = 3).

In the next step, the dissolution rate of baicalin from the F1–F6 formulation was assessed (Figure 7). As described above, an increased dissolution rate of baicalin from the lyophilized extract and extrudates was observed, which is related to the change from crystalline to amorphous form (Figure 4). The dissolution profiles of baicalin from extrudates and formulations prepared from them (extrudates 75:25 and F1, extrudates 50:50 and F3, and extrudates 25:75 and F5) differing in the pressure used to prepare the tablets were compared. In each case, it was noticed that the dissolution rate decreased with the increase in compression pressure, but the differences were not statistically significant (in all cases, *f*_1_ was below 20 and *f*_2_ was above 50). Importantly, baicalin release from extrudate-based formulations was relatively fast, even faster than release from powder-based tablets. In the case of powder-based tablets, a slow and controlled release was observed (Figure 7d–f). This substantial difference can be explained by the swelling behavior of these extrudates as well as HPMC, calculated from equation no. 7 (Figure 8). While powder-based systems rapidly absorb water upon contact with the release medium, extrudate-based tablets remained almost intact. The HPMC powder swelled significantly, and the active compounds had to pass through the polymer network, and a more extensive layer of gel formed around the powder tablets, which made it difficult for baicalin to dissolve and enter the release medium [22]. HME changes the behavior of HPMC, and the reprocessed carrier has less water absorption and sticky layer properties. In addition, changes in the release of baicalin can be observed depending on the amount of HPMC in the system. When the percentage of HPMC in the formulation increased, the baicalin release rate decreased simultaneously, both from extrudate- and powder-based tablets.

Differences in the release of baicalin from extrudate- and powder-based tablets are also visible in the kinetics of its release. Mathematical models describing the release kinetics of baicalin from formulations F1–F6 are collected in Table S2 (Supplementary Materials). As indicated above, the release of baicalin from powder-based tablets (formulations F2, F4, and F6) is considerably slowed down and controlled, and the release of baicalin follows zero-order kinetics. It means that the release rate of baicalin is constant over a period of time. Such controlled release systems are indicated and developed in therapeutic drug delivery systems, but if mucosal application within the oral cavity is desired, complete release of the substance should occur within 2 h because a longer stay of the tablet stuck to the mucous membrane may be uncomfortable for the patient. In this regard, baicalin release is preferred from an extrudate-based tablet (formulations F1, F3, and F5). For these formulations, as the most probable, the Higuchi model was shown, which best describes the release from the matrix system and suggests that the baicalin was primarily released by diffusion and that its release was from a homogeneous flat matrix that did not degrade [14]. Additionally, a good fit to Korsmeyer-Peppas with ‘n’ values in the range 0.45–0.89 indicated the release approximated the non-Fickian diffusion release mechanism [23]. The relative complexity of the prepared formulations may indicate that the active compound release is controlled by more than one mechanism; a coupling of polymer erosion, swelling, and dissolution, which were all involved in the release process, which is consistent with the previous literature data [24].

**Figure 9:**
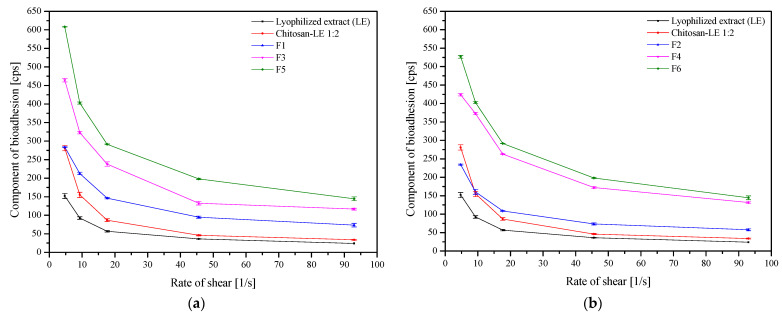
Component of bioadhesion of the extrudate-based tablets (**a**) and the powder-based tablets (**b**) (*n* = 3).

Finally, the mucoadhesive properties of formulations F1–F6 were evaluated by rheological measurements (Figure 9). The basis of the blends is a lyophilized extract with chitosan, to which HPMC was added as a carrier. The mucoadhesive properties of chitosan are widely known, and chitosan–mucin interact mainly electrostatically, supported by other types of interactions (e.g., hydrogen bonds and hydrophobic association) [25]. In this case, to demonstrate the mucoadhesive effect, an appropriate pH is necessary (pH < 6), so the pH of the oral cavity is on the limit [26]. However, HPMC is a non-ionic polymer; the medium’s pH had no effect on how well it stuck to the mucosa. Thus, in the case of the described blends, HPMC is the primary mucoadhesive agent. It has a lot of hydroxyl groups, which allow it to form intermolecular bonds (including hydrogen interactions) with the components of mucus [27,28]. Formulations containing unprocessed HPMC, i.e., powder-based blends (formulations F2, F4, and F6) had more vital adhesion forces than their corresponding extrudate-based blends, possibly due to their elasticity, hydrogen bonding, molecular weight, and cross-linking. Internal forces are represented by viscosity, whereas the force needed to separate a polymer from a surface is known as adhesion force [28]. Moreover, the adhesive force of all blends decreased with a decrease in the HPMC content, which aligns with previous outcomes [29,30].

Tablets were additionally tested for their residence time to elaborate on their mucoadhesive behavior upon continuous contact with the medium-simulating saliva (Table 3). All of the formulations that were tested attached to the tissue right away, swelled progressively when they came into contact with the acceptor medium, and showed no evidence of disintegration at any point during the test. Despite the continuous movement of the cylindrical probe, the contact time of tablets F4 and F6 with the mucosal surface was preserved within 240 min of the test. In contrast, formulations F2 and F5 separated from the tissue after 220 min, F3 after 200 min, and F1 after 180 min (Table 3). This behavior of the tablets may be due to the higher viscosity and greater mucoadhesive strength of the unprocessed HPMC, as described above.

## 3. Materials and Methods

### 3.1. Plant Material

Plant raw material, *Scutellariae baicalensis radix*, was purchased from NANGA (Zlotow, Poland), the country of origin: China (Lot No. 243042021).

### 3.2. Chemicals and Reagents

Baicalin (≥95%, HPLC) was obtained from Sigma-Aldrich (Poznan, Poland). Excipients, such as chitosan with a degree of acetylation of 90% with a viscosity range of 500 mPas (marked as 90/500), was supplied from Heppe Medical Chitosan GmbH (Halle, Germany), (hydroxypropyl)methyl cellulose (HPMC) with an average Mn~90.000 (~15.000 cP), and magnesium stearate, were supplied by Sigma-Aldrich (Poznan, Poland). Microcrystalline cellulose (MCC) VIVAPUR 102 was supplied by JRS PHARMA (Rosenberg, Germany). Reagent for mucoadhesive tests: mucin from porcine stomach was obtained from Sigma-Aldrich (Poznan, Poland). HPLC grade acetonitrile and water were obtained from Merck. High-quality pure water and ultra-high-quality pure water were prepared using an Direct-Q 3 UV Merck Millipore purification system.

### 3.3. Preparation of Solid Dispersion Systems

#### 3.3.1. Preparation of Extract System

5.0 g of the dried root of *Scutellariae baicalensis radix* was extracted four times with an ethanol–water mixture (8:2 *v*/*v*) for 60 min at 70 °C on an ultrasound-assisted water bath. The obtained extracts were collected and concentrated on a vacuum evaporator at a temperature 50°C to a volume of 20.0 mL (BÜCHI Rotavapor R-210) obtaining at that time DER 1:4. Then the extract was frozen and lyophilized (CHRIST 1–4 LSC, Osterode am Harz, Germany). The temperature on the freeze dryer shelf was heated and ranged from +15 °C to +20 °C; the temperature inside the product was estimated −4 °C; and the condensation temperature was set at −48 °C. The freeze-drying was conducted at reduced pressure (1.030 mbar) for 48 h. So obtained lyophilized extract was combined with chitosan 90:500 in a weight ratio of 2:1 and named as ‘system’ for further tests [14].

#### 3.3.2. Hot Melt Extrusion (HME)

Extrusion was performed on a HAAKE MiniCTW micro-conical twin screw extruder (Thermo Scientific, Karlsruhe, Germany). The above-described system of lyophilized extract and chitosan in ratio 1:2 and carrier (HPMC) in three different ratios (Table 4) were mixed with a mortar and pestle and subsequently fed manually into the hopper of the extruder at barrel temperature of 150 °C and screw speed of 150 rpm. The extrudates were collected, ground softly manually with a pestle and mortar, passed through an 80 mesh sieve, and kept in a desiccator at room temperature for further analysis.

#### 3.3.3. Extrudate Characterization

##### Powder X-ray Diffraction (PXRD)

The crystallographic structure of the samples was analyzed by X-ray diffraction (XRD, Panalytical Empyrean, Almelo, The Netherlands) equipment with the copper anode (CuKα—1.54 Å) in a Brag-Brentano reflection mode configuration with 45 kV and 40 mA parameters. The measurement parameters were set up for 3–60° with a 45 s per step 0.05° in all cases.

##### Fourier Transform Infrared Spectroscopy with Attenuated Total Reflectance (FTIR-ATR)

The FTIR-ATR spectra were measured between 400 cm^−1^ and 4000 cm^−1^, with a resolution set to 1 cm^−1^, with a Shimadzu IRTracer-100 spectrometer equipped with a QATR-10 single bounce—diamond extended range—and LabSolution IR software.

#### 3.3.4. Determinations of Active Components Content

The contents of the main active compounds (baicalin, baicalein, and wogonin) were determined by using the HPLC-Diode-Array Detection method described previously by Paczkowska-Walendowska et al. [14]. Briefly, separations were performed on a Kinetex^®^ C18 column, 5 μm particle size, 100 mm × 2.1 mm (Phenomenex, Poland). The mobile phase was composed of phosphoric acid 0.1% (A) and acetonitrile (B), with a gradient elution: 0–20 min, 10–40% B; 20–22 min, 10% B. The detection was performed at a wavelength (λ_max_) of 280 nm. The flow rate of the mobile phase was set at 1.0 mL/min, and the column temperature was set at 30 °C. Injection volume was 10 µL. The test was repeated three times.

#### 3.3.5. In Vitro Release Studies

An Agilent 708-DS apparatus was used for the dissolution studies. At 37 ± 0.5 °C, a typical paddle method was employed, with 50 rpm for stirring. Extrudates samples (~100 mg) were dissolved in 300 mL of an artificial saliva solution with the following ingredients: potassium chloride (1.20 g), sodium chloride (0.85 g), dipotassium hydrogen orthophosphate (0.35 g), magnesium chloride (0.05 g), calcium chloride (0.20 g), xylitol (20.0 g), and water up to 1L; the pH was adjusted to 6.8 by 1 M HCl. At certain intervals (15, 30, 60, 120, and 240 min), liquid samples were taken, and an equal volume of temperature-stabilized medium was substituted. A nylon membrane filter with a mesh size of 0.45 was used to filter the samples. The previously published HPLC method was used to ascertain the levels of baicalin in the filtered acceptor solutions. Sink conditions were preserved in the studies. The test was repeated for six samples of each substance.

The release profiles were compared by means of the model proposed by Moore and Flanner, which is based on two-factor values, *f*_1_ and *f*_2_.

#### 3.3.6. Permeability Studies

The permeability of an active compound (baicalin) enclosed in systems through artificial biological membranes was investigated by using the PAMPA™ (parallel artificial membrane permeability assay) gastrointestinal tract (GIT) assay (Pion Inc., Billerica, MA, USA) according to the protocol supplied with the kit. Extrudate- and powder-based systems (concentration 10 mg/mL) were dissolved in donor solutions (artificial saliva solution at pH 6.8). The acceptor plates were loaded with acceptor Prisma buffer at pH 7.4. The plates were put together and incubated under the following conditions: temperature set at 37 °C for 15 min with continuous stirring at 50 rpm. Each experiment was repeated at least three times. The amount of permeated baicalin was determined using the HPLC method described above. The test was repeated six samples of each substance. The apparent permeability coefficients (P_app_) were calculated from the following equation:

(1) $$P_{app}=\frac{-ln\left(1-\frac{C_{A}}{C_{equilibrium}}\right)}{S\times \left(\frac{1}{V_{D}}+\frac{1}{V_{A}}\right)\times t}$$

where *V_D_* is the donor volume, *V_A_* is the acceptor volume, *C_equilibrium_* is the equilibrium concentration $C_{equilibrium}=\frac{C_{D}\times V_{D}+C_{A}\times V_{A}}{V_{D}+V_{A}}$, *C_D_* is the donor concentration, *C_A_* is the acceptor concentration, *S* is the membrane area, and *t* is the incubation time (in seconds).

#### 3.3.7. Microbiological Activity Assay

##### Well Diffusion Method

All microorganism strains were inoculated in Müeller-Hinton broth (pH 7.4) for approximately 16 h. The concentration of the suspensions was adjusted to 0.5 (optical density) by means of a spectrophotometer. Antimicrobial activity of the *S. baicalensis radix* lyophilized extract and extrudates were determined by the Agar well diffusion method against reference strains and clinical isolates of bacteria that colonize the oral cavity (*Escherichia coli, Pseudomonas aeruginosa, Streptococcus mutans, Staphylococcus aureus, Staphylococcus epidermidis,* and *Enterobacter aerogenes*). The 20 mL of sterilized Nutrient Agar was poured into sterile petri plates. Following solidification, 100 μL of standardized inoculate from each isolate was inoculated on Nutrient agar plates using sterilized spreaders. The wells were punched over the agar plates using a sterile gel puncher of 6 mm diameter. A measure of 100 μL of the lyophilized extract and extrudates was poured into separate wells. Samples were dissolved in 1% (*v*/*v*) dimethylsulphoxide (DMSO), which was used as a negative control. Plates were incubated at 37 °C for 24 h. Triplets of the experiment were maintained for each bacterial strain to ensure reliability. Following incubation, the diameter of the circular inhibitory zones formed around each well was measured in mm and recorded.

##### Liquid Culture Method

In the first stage of the research, strains of microorganisms were prepared. For this purpose, 0.1 g of bacterial lyophilisate was suspended in 10 mL of Müeller-Hinton liquid propagation medium. The samples were incubated at 37 °C for 18 h in order to activate and multiply the biomass. After incubation, the biomass was centrifuged from the substrate (14.000 rpm for 10 min). The supernatant was discarded, and the pellet was resuspended in 10 mL of 0.9% NaCl and centrifuged again. This procedure was performed three times. Then, the biomass was diluted in 0.9% NaCl, so that the concentration of microorganisms was 1.0 × 10^2^ cfu/mL. At the same time, three solutions were prepared (the solvent was 0.9% NaCl) of the test samples at a concentration of 10, 50, and 100 mg/mL. Then, the dilutions prepared in this way were inoculated with the prepared suspension of microorganisms. The samples were mixed and incubated at 37 °C for 18 h. The number of microorganisms was analyzed before and after incubation using media intended for a given group of microorganisms.

### 3.4. Tableting Process

A laboratory scale, single-punch tableting equipment called the NP-RD10A Tablet Press was used to compressed tablets that were flat-faced and 8 mm in diameter (Natoli, Saint Charles, MO, USA). Utilizing a variety of compaction forces between 1000 and 3000 N, the compaction characteristics of tablets were evaluated (corresponding to compression pressures in a range from 20 to 60 MPa). When the desired compaction force was reached, the pressure was let go. Two types of formulations were prepared, containing extrudates or powder systems in appropriate proportions of ingredients. Table 5 lists the ingredients of the formulations.

#### 3.4.1. Tablet Characterization

Immediately following the tablets compacting, the newly created tablets were weighed. A procedure outlined in Ph.Eur. 9th was used to control the uniformity of the tablet mass. A manual vernier caliper was also used to measure the diameter and thickness of 20 tablets that were chosen at random. Standard deviations and mean values were computed following all measurements (SD).

The tablet hardness was determined using the procedures outlined in Ph.Eur. 9th and was evaluated using the PTB-M manual tablet hardness testing device (Natoli, Saint Charles, MO, USA). Each hardness number is a mean with a standard deviation that represents the average of six measurements.

Tensile strength (*σ*) values were calculated on the basis of the breaking force (*F*) values (N), where d is the diameter of the tablet (mm) and h is the thickness of the tablet (mm) [31].

(2) $$\sigma =\frac{2F}{\pi dh}$$

Solid fraction (*SF*) was calculated by the equation, where *W_t_* is the weight of the tablet (mg), v is the tablet volume, and *ρ_true_* is the powder true density (g/cm^3^).

(3) $$F=\frac{W_{t}}{\rho_{true}v}$$

The tablet porosity (*ε*) was calculated from the SF using the following equation:

(4) ε=1−SF

Compactibility of the powders were analysed with the Ryshkewitch equation:

(5) $$\varepsilon =\varepsilon_{0}\times \exp\left(-b\times P\right)$$

(6) $$T_{S}=T_{0}\times \exp\left(-k\times \varepsilon \right)$$

where the porosity of powder when *p* = 0; *b* is a constant that is inversely proportional to the yield strength of the materials; *T_S_* and *T*_0_ are the tablet tensile strength and the limiting tablet tensile strength at zero porosity, respectively, and k is an empirical constant [19].

#### 3.4.2. In Vitro Release Studies

In vitro release studies were performed according to the methodology described in Section 3.3.5. The test was repeated 6 times for each formulation.

The resulting active compound release profiles were fitted to the following mathematical models in order to study the release kinetics: [32]: zero-order equation: F=k×t, first-order equation: nF=k×t, Higuchi equation: $F=kt^{1/2}$, Korsmeyer-Peppas equation: $F=kt^{n}$, where *F*—the fraction of released drug, *k*—the constant associated with the release, and *t—*the time.

#### 3.4.3. Swelling Index

Each tablet was individually weighted and placed in a 25 mL beaker that contained 10 mL of an artificial saliva solution at pH of 6.8 and at 37 ± 0.5 °C. Tablets were taken out, cleaned with filter paper, and reweighted at the preset intervals (15, 30, 60, 120, and 240 min). The swelling index was calculated by using the following formula:

(7) $$SI=\frac{W_{2}-W_{1}}{W_{1}}$$

where *SI* is the swelling index, *W*_1_ is the initial weight of the tablet, *W*_2_ is the weight of the tablet after the particular swelling time interval.

Each experiment was performed in triplicate.

#### 3.4.4. In Vitro Assessment of Mucin-Biopolymer Bioadhesive Bond Strength

A viscometric method was used to quantify mucin-polymers’ bioadhesive bond strength. The assessment was carried out according to Hassan and Gallo’s procedure [33]. Each experiment was performed in triplicate.

#### 3.4.5. Determination of the Residence Time

The residence time of tablets on regenerated cellulose membranes imitating porcine buccal mucosa was evaluated on an adjusted apparatus for the disintegration time test according to previous tests described by Paczkowska-Walendowska et al. [29]. Briefly, the medium was an artificial saliva solution at pH 6.8 maintained at 37 ± 0.5 °C. Each tablet was brought into contact with foil by putting on a finger force for 5 s. The time necessary to detach the formulation from the foil simulating mucosal tissue was measured within 4h of the performed test. Studies were carried out in triplicate.

### 3.5. Statistical Analysis

Software called Statistica 13.3 was used for the statistical analysis. The Shapiro-Wilk test was used to determine whether the results were normal. The ANOVA test, together with the post hoc Tukey’s range test for multiple comparisons, was used to examine the variances between the mean values. At *p* < 0.05, differences between groups were deemed significant.

## 4. Conclusions

Extrudates containing *Scutellariae baicalensis radix* extract can be prepared at temperatures of about 150 °C, which does not decompose the active compounds. The proposed ground hot-melt extrudates based on HPMC show an interesting potential for improving the solubility of the poorly water-soluble active substance—baicalin. So, hot-melt extrusion is a good technique to improve the physicochemical properties of baicalin. Further, in order to obtain a suitable pharmaceutical form, the production process of mucoadhesive tablets containing extrudates was optimized. The prepared extrudates, differing in HPMC content, showed different tabletability, compressibility, and compactibility properties. As expected, the different content of the carrier influenced the release profile of baicalin from the tablets and the mucoadhesive properties. Higher HPMC content resulted in prolonged release of the substance, resulting from the diffusion of the substance through the polymer network. At the same time, the same carrier ensured that the tablets were kept in the affected area for a sufficiently long time. Importantly, the process did not reduce the biological, including microbiological, activity of the obtained extrudates.

Considering the complex matrix, both the tabletability/compactibility properties of the blends and the degree of release of the active substance, as well as mucoadhesive properties that give functionality to the developed tablets, should be considered. The best tabletability properties, a valuable baicalin release profile while maintaining sufficient mucoadhesive properties to condition the tablet’s retention in the application site and the effectiveness of therapy, are provided by the F3 formulation, which contains the extrudate with lyophilized extract-HPMC 50:50 *w*/*w*.

## Figures and Tables

**Table 1:** XRPD Signals’ Positions.

Sample	Lyophilized Extract	Chitosan	HPMC	System-HPMC Extrudate 75:25	System-HPMC Extrudate 50:50	System-HPMC Extrudate 25:75
(1) Peak position [2*θ*]	-	11.14	9.77	-	-	-
(2) Peak position [2*θ*]	23.12	20.19	19.83	20.36	20.51	20.27
Matrix peak position displacement [2*θ*]	-	-	-	- (2) 0.17	- (2) 0.32	- (2) 0.08
Matrix peak position displacement [Å]	-	-	-	- (2) −0.03	- (2) −0.07	- (2) −0.02
(1) Peak position [2*θ*]	-	11.14	9.77	-	-	-
(2) Peak position [2*θ*]	23.12	20.19	19.83	20.36	20.51	20.27
Matrix peak position displacement [2*θ*]	-	-	-	- (2) 0.17	- (2) 0.32	- (2) 0.08

**Table 2:** Influence of the raw material on indicator microorganisms (reference strains and clinical isolates) by the liquid culture method—concentration of the test sample 100 mg/mL.

	Lyophilized Extract	Extrudate 25:75	Extrudate 50:50	Extrudate 75:25
	Number of microorganisms [CFU]			
*Escherichia coli* ATCC13706	6.9 × 10^2^ → 3.9 × 10^7^	4.8 × 10^2^ → nd	2.0 × 10^2^ → 3.9 × 10^5^	2.0 × 10^2^ → 1.4 × 10^7^
*Escherichia coli*—clinical isolate	3.4 × 10^2^ → 5.1 × 10^7^	2.0 × 10^2^ → nd	3.3 × 10^2^ → 8.0 × 10^4^	2.9 × 10^2^ → 3.3 × 10^7^
*Pseudomonas aeruginosa* ATCC27853	2.1 × 10^2^ → 5.9 × 10^7^	2.7 × 10^2^ → nd	4.4 × 10^2^ → 3.7 × 10^5^	2.0 × 10^2^ → 5.8 × 10^6^
*Pseudomonas aeruginosa*—clinical isolate	2.5 × 10^2^ → 6.0 × 10^6^	2.0 × 10^2^ → nd	5.8 × 10^2^ → 3.6 × 10^5^	2.0 × 10^2^ → 5.3 × 10^7^
*Streptococcus mutans* ATCC25175	6.9 × 10^2^ → 3.9 × 10^7^	4.8 × 10^2^ → nd	2.0 × 10^2^ → 3.9 × 10^5^	2.0 × 10^2^ → 1.4 × 10^7^
*Streptococcus mutans*—clinical isolate	3.4 × 10^2^ → 5.1 × 10^7^	2.0 × 10^2^ → nd	3.3 × 10^2^ → 8.0 × 10^5^	2.9 × 10^2^ → 3.3 × 10^7^
*Staphylococcus aureus* ATCC 6538	2.6 × 10^2^ → 3.0 × 10^7^	3.6 × 10^2^ → nd	2.9 × 10^2^ → 1.7 × 10^4^	1.9 × 10^2^ → 5.9 × 10^6^
*Staphylococcus aureus*—clinical isolate	2.0 × 10^2^ → 3.7 × 10^7^	1.9 × 10^2^ → nd	2.6 × 10^2^ → 3.0 × 10^5^	3.4 × 10^2^ → 3.0 × 10^6^
*Staphylococcus epidermidis* NCTC 11047	3.6 × 10^2^ → 3.3 × 10^6^	2.0 × 10^2^ → nd	2.5 × 10^2^ → 3.2 × 10^4^	2.1 × 10^2^ → 3.6 × 10^7^
*Staphylococcus epidermidis*—clinical isolate	7.7 × 10^2^ → 8.5 × 10^4^	2.9 × 10^2^ → 3.3 × 10^2^	3.4 × 10^2^ → 5.1 × 10^4^	2.9 × 10^2^ → 3.0 × 10^8^
*Enterobacter aerogenes* ATCC 51697	2.6 × 10^2^ → 3.0 × 10^5^	3.6 × 10^2^ → 3.0 × 10^2^	2.9 × 10^2^ → 1.7 × 10^5^	1.9 × 10^2^ → 5.9 × 10^6^
*Enterobacter aerogenes*—clinical isolate	2.0 × 10^2^ → 3.7 × 10^6^	1.9 × 10^2^ → nd	2.6 × 10^2^ → 3.0 × 10^4^	3.4 × 10^2^ → 3.0 × 10^6^

nd—no detected.

**Table 3:** The residence time of the extrudate- and powder-based tablets (*n* = 3).

Formulation	F1	F2	F3	F4	F5	F6
Residence time (min)	180 ± 5	220 ± 5	200 ± 5	>240	220 ± 5	>240

**Table 4:** Compositions of extrudates.

	Chitosan 90/500—Lyophilized Extract 1:2 (=System)	HPMC
	Ratio (*w*/*w*)	
Extrudate 75:25 *m*/*m*	75	25
Extrudate 50:50 *m*/*m*	50	50
Extrudate 25:75 *m*/*m*	25	75

**Table 5:** Compositions of formulations.

	Formulation 1 (F1)	Formulation 2 (F2)	Formulation 3 (F3)	Formulation 4 (F4)	Formulation 5 (F5)	Formulation 6 (F6)
	Content (mg) per 1 tablet					
Lyophilized extract—chitosan 90/500 2:1 *w*/*w* (=system)	-	75.0	-	50.0	-	25.0
HPMC 15.000 cP	-	25.0	-	50.0	-	75.0
System-HPMC 75:25 *w*/*w* extrudate	100.0	-	-	-	-	-
System-HPMC 50:50 *w*/*w* extrudate	-	-	100.0	-	-	-
System-HPMC 25:75 *w*/*w* extrudate	-	-	-	-	100.0	-
MCC	20.0	20.0	20.0	20.0	20.0	20.0
Stearate magnesium	1.2	1.2	1.2	1.2	1.2	1.2
SUM	121.2	121.2	121.2	121.2	121.2	121.2

## Data Availability

The data are contained within the article and Supplementary Materials.

## Supplementary Materials

The following supporting information can be downloaded at: https://www.mdpi.com/article/10.3390/ijms24065834/s1.
