# Supplementary material for: Hot Melt Extrusion as an Effective Process in the Development of Mucoadhesive Tablets Containing Scutellariae baicalensis radix Extract and Chitosan Dedicated to the Treatment of Oral Infections
Source: Int J Mol Sci. 2023 Mar 19;24(6):5834. doi: 10.3390/ijms24065834 (PMC10054152; doi:10.3390/ijms24065834)
Supplement: Supplementary file 1 [file ijms-24-05834-s001.zip › ijms-2270785-supplementary.pdf]

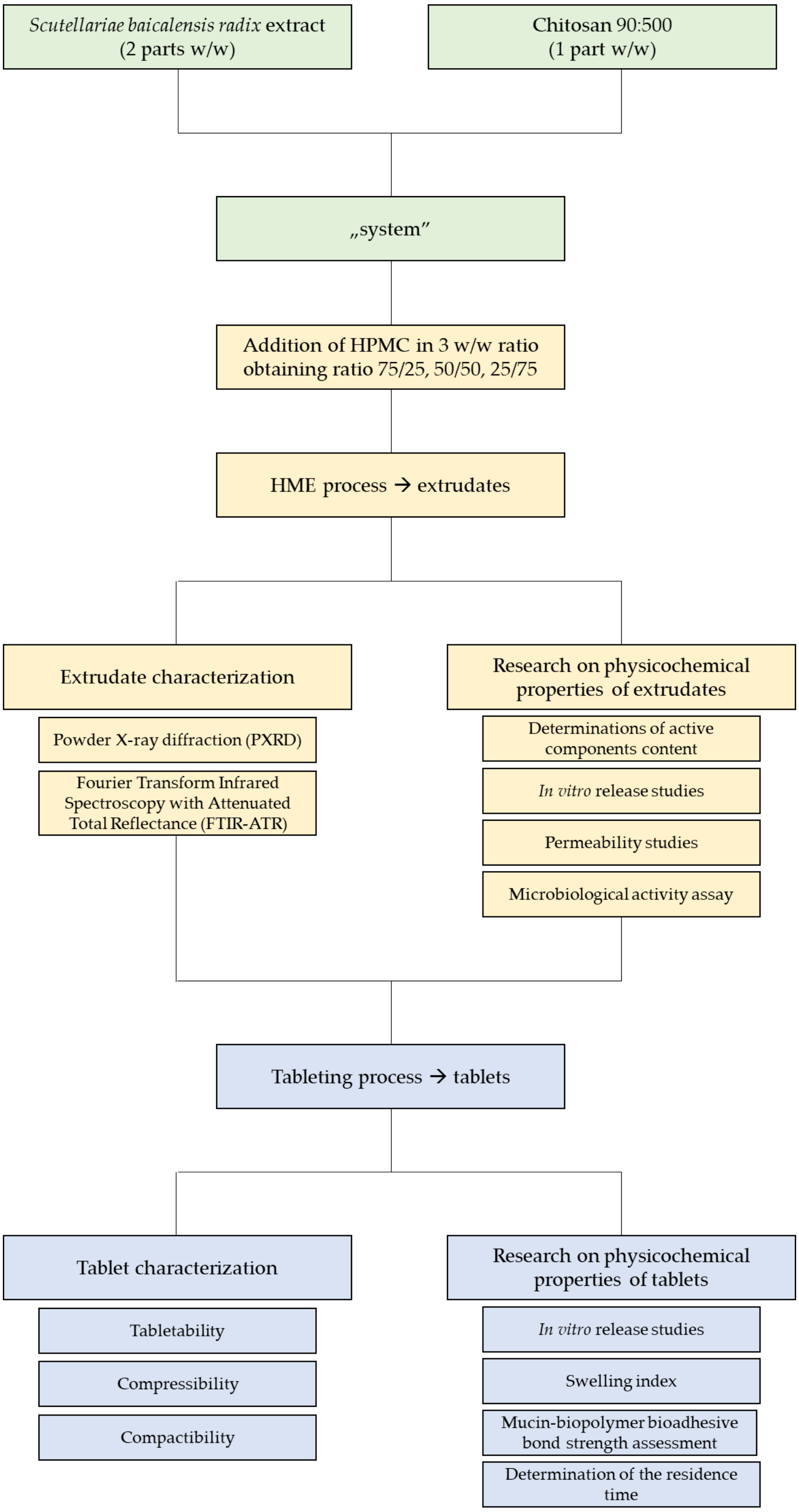

Figure S1. Flow chart of all experimental works (stages of system development are marked in green, stages of HME process development are marked in yellow, and stages of tableting process development are marked in blue).

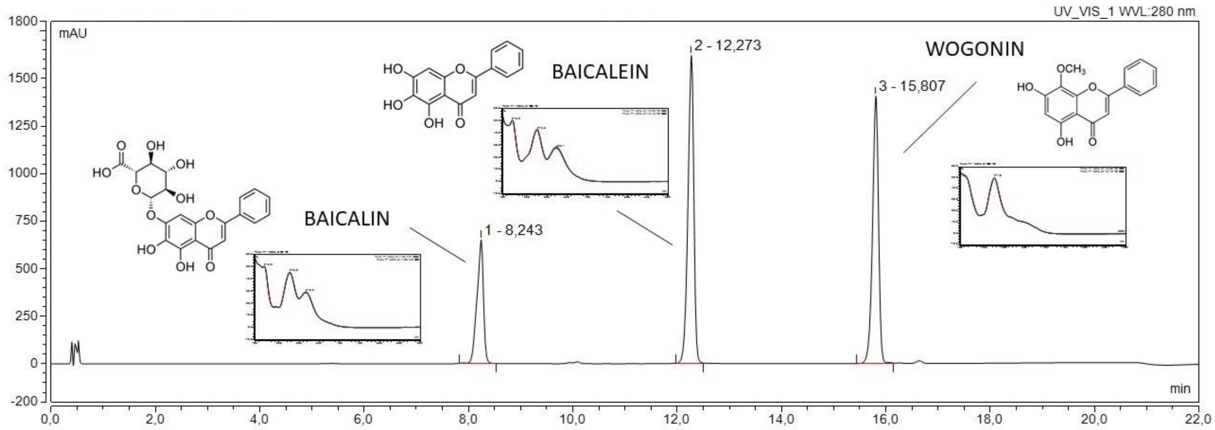

Figure S2. Chromatogram of standards.

Table S1. Validation parameters of HPLC method

Parameter	**Baicalin**	**Baicalein**	**Wogonin**
Linearity: y = ax + b			
$a \pm S_a$	0.5449 ± 0.0218	0.7326 ± 0.0214	1.6129 ± 0.0321
$b \pm S_b$	insignificant (α=0.05)	insignificant (α=0.05)	insignificant (α=0.05)
Correlation coefficient (r)	0.9984	0.9991	0.9996
Range of linearity [mg/mL]	0.0520–5.200	0.0870–0.8700	0.0320–0.3200
Limit of detection (LOD) [μg/mL]	48.0278	58.7691	14.7156
Limit of quantification (LOQ) [μg/mL]	145.5388	178.0883	44.5927

Table S2. Mathematical models of release profiles of formulations F1-F6

Formulation		Mathematical model							
No.	Compression pressure	Zero-order kinetic		First-order kinetic		Higuchi kinetic		Korsmeyer-Peppas kinetic	
		K	R^2	K	R^2	K	R^2	n	R^2
F1	20 MPa	21.01	0.72	0.67	0.33	**18.50**	**0.97**	0.77	0.84
	40 MPa	19.76	0.73	0.66	0.34	**17.34**	**0.97**	0.77	0.83
	60 MPa	20.91	0.79	0.71	0.40	**17.09**	**0.98**	0.82	0.83
F2	20 MPa	**24.59**	**0.97**	0.81	0.55	16.52	0.90	**0.90**	**0.83**
	40 MPa	**23.82**	**0.97**	0.84	0.60	15.64	0.88	**0.93**	**0.83**
	60 MPa	**24.13**	**0.99**	0.87	0.65	15.18	0.84	**0.94**	**0.82**
F3	20 MPa	21.23	0.72	0.69	0.36	**18.51**	**0.97**	0.79	0.84
	40 MPa	21.62	0.79	0.70	0.38	**17.86**	**0.98**	0.80	0.84
	60 MPa	22.63	0.83	0.74	0.42	**17.76**	**0.97**	0.84	0.85
F4	20 MPa	**24.62**	**0.94**	0.84	0.57	16.80	0.91	**0.92**	**0.85**
	40 MPa	**23.85**	**0.95**	0.83	0.57	16.15	0.90	**0.92**	**0.84**
	60 MPa	**23.00**	**0.97**	0.83	0.58	15.40	0.90	**0.92**	**0.83**
F5	20 MPa	21.79	0.76	0.70	0.37	**18.33**	**0.98**	0.80	0.85
	40 MPa	21.93	0.82	0.72	0.40	**17.49**	**0.98**	0.82	0.84
	60 MPa	21.16	0.89	0.79	0.49	**17.76**	**0.95**	0.88	0.85
F6	20 MPa	**24.53**	**0.94**	0.84	0.57	16.82	0.91	**0.92**	**0.85**
	40 MPa	**23.82**	**0.94**	0.93	0.57	16.32	0.91	**0.92**	**0.84**
	60 MPa	**22.80**	**0.96**	0.82	0.57	15.41	0.91	**0.92**	**0.83**
